# LINE-1 and Alu retrotransposition exhibit clonal variation

**DOI:** 10.1186/1759-8753-4-16

**Published:** 2013-06-03

**Authors:** Vincent A Streva, Zachary J Faber, Prescott L Deininger

**Affiliations:** 1 Graduate Program in Biomedical Sciences, Tulane University Health Sciences Center, New Orleans, LA 70112, USA; 2 Department of Epidemiology, Tulane Cancer Center, Tulane University School of Public Health and Tropical Medicine, New Orleans, LA 70112, USA; 3 St Jude Children’s Research Hospital, Memphis, TN 38105, USA

**Keywords:** LINE-1, Alu, Retrotransposition, Clone, Variation

## Abstract

**Background:**

The non-long terminal repeat (non-LTR) retrotransposons, long interspersed element-1 (LINE-1) and Alu are currently active retroelements in humans. We, and others, have observed that different populations of HeLa cells from different laboratories support retrotransposition of LINE-1 and Alu to varying degrees. We therefore tested whether individual cell clones of HeLa and HCT116 cell lines supported different levels of LINE-1 and Alu retrotransposition, and whether these variations were stable upon re-cloning.

**Findings:**

Standard retrotransposition tissue culture assays were used to measure a cell’s ability to support LINE-1 and Alu retrotransposition in clonal HeLa and HCT116 cell lines. We observed that both LINE-1 and Alu retrotransposition exhibited clonal variation in HeLa cells, with certain HeLa cell clones supporting high levels of LINE-1 and Alu retrotransposition and other cell clones being essentially retrotransposition-dead. This clonal variation was similarly observed in HCT116 cells, although possibly not to the same extent. These patterns of clonal variation are relatively consistent upon re-cloning.

**Conclusions:**

Observations of the variability of LINE-1 and Alu retrotransposition in different populations of the same cell line are supported by our results that indicate in some cell types, individual cell clones can have dramatically differing capacity for retrotransposition. The mixed populations of cells commonly used in laboratories have often been passaged for many generations and accumulated significant genetic and epigenetic diversity. Our results suggest that the clonal variability observed by our cloning experiments may lead to a homogenization of retrotransposition capacity, with the resulting mixed population of cells being composed of individual variants having either increased or decreased retrotransposition potential compared to the starting population.

## Findings

### Introduction

Long interspersed element-1 (LINE-1) and Alu retrotransposons make up nearly one half of the DNA content of the human genome [1]. Mobilization of autonomous LINE-1 and non-autonomous Alu elements is currently ongoing in human genomes and has been implicated in a number of genetic diseases [2-4]. In order to study mobilization of LINE-1 and Alu, plasmid-based reporter systems have been widely used [5]. Retrotransposition rates appear to vary widely between different cell types, but it has also been observed that different populations of the same cell type support LINE-1 and Alu retrotransposition to varying degrees [6]. Additionally, we have observed fluctuation in the retrotransposition potential of cell lines as they are grown in our laboratory over time and others have observed differential effects on LINE-1 and Alu within variations of HeLa [6]. We wished to determine whether this variation was at least partially due to genetic variation and evolution of cells in culture. These observations led us to compare the potential for LINE-1 and Alu retrotransposition of clones of two commonly used cell lines, HeLa and HCT116.

## Methods

HeLa and HCT116 cells were obtained from ATCC (Manassas, VA, USA). HeLa and HCT116 cells were maintained in minimum essential medium (MEM) (Invitrogen, Carlsbad, CA, USA) supplemented with 10% fetal bovine serum (FBS; Gibco, Invitrogen), non-essential amino acids and sodium pyruvate (Invitrogen). HeLa and HCT116 cell lines were cloned by limiting dilution in a 96-well plate format and multiple clones were tested for LINE-1 and Alu retrotransposition using previously described reporter assays [5].

Briefly, one million cells were seeded per 75 cm^2^ flask. One day after seeding, cells were transfected using Lipofectamine (Invitrogen) according to the manufacturer’s protocol, with either 1 μg JM101/L1.3 [5] for LINE-1 retrotransposition assays or 1 μg AluYa5-neo^TET^[7] and 1 μg JM101/L1.3-no tag [8] (an untagged expression cassette for L1.3, provided by JV Moran) as a driver for Alu retrotransposition assays. The plasmids, upon integration, reverse transcription and retrotransposition, yield cells resistant to the antibiotic G418, allowing measurement of the rate of LINE-1 and Alu retrotransposition. In parallel, to control for transfection efficiency, which can vary between cell clones and between experiments, clones were transfected with 300 ng of pIRES (Addgene, Cambridge, MA, USA), a plasmid that confers G418 resistance to the transfected cells.

Twenty-four hours after transfection, media containing G418 was added to select for integration events. G418 selection was maintained for 10 to 14 days until visible colony formation. Colonies were stained with 5% (w/v) crystal violet solution and counted using an automated cell counter (ColCount, Oxford Optronix, Abingdon, UK). Retrotransposition colony counts were normalized to pIRES colonies for each experiment to correct for differences in transfection efficiency. For each independent experiment, the clone with the highest transfection efficiency, as determined by pIRES colony number, was set as 100% and each other clone was adjusted by the percentage that that clone differed from the clone with the highest transfection efficiency for that experiment. The mean number of pIRES colonies across all experiments was 1,050 (SD = 332). Data are presented for LINE-1 and Alu retrotransposition. Statistical significance was performed using GraphPad Prism software (La Jolla, CA, USA) and one-way ANOVA with Tukey’s post-test to compare means.

## Results

**Figure 1:**
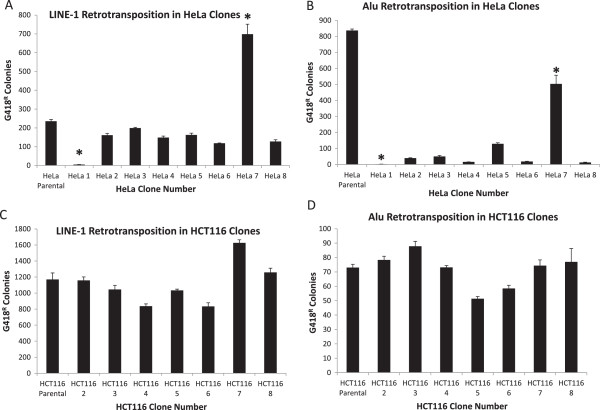
**LINE-1 and Alu exhibit clonal variation. (A)** LINE-1 retrotransposition assays were performed in eight individual HeLa cell clones and parental HeLa population. Different HeLa clones exhibit different potential for LINE-1 retrotransposition, with HeLa clone 1 exhibiting a 140-fold reduction in LINE-1 retrotransposition rate compared to HeLa clone 7. **(B)** Alu retrotransposition assays were performed in HeLa cell clones and parental HeLa. Differences in Alu retrotransposition between the two HeLa clones were even greater than those observed for LINE-1, with a 252-fold reduction in Alu retrotransposition in HeLa clone 1 compared to HeLa clone 7. **(C)** LINE-1 retrotransposition assays were also preformed in HCT116 cell clones to examine whether the effect seen in HeLa also occurred in HCT116 cells. The LINE-1 retrotransposition clonal variation was not observed in five HCT116 cell clones. **(D)** Alu retrotransposition assays were tested in seven HCT116 cell clones and parental HCT116 cell population and no significant differences in rates of Alu retrotransposition were observed between HCT116 clones. Error bars represent SEM. Sample size is four independent experiments (independent transfections) each performed in triplicate (n = 12). Asterisks indicate statistical significance from the parental population of at least *P* <0.05 by one-way ANOVA with Tukey’s post-test. LINE-1, long interspersed element-1; SEM, standard error of the mean.

Because many cancer cell lines have relatively unstable genomes, we hypothesized that their genome variation could lead to altered abilities to support retrotransposition. To determine whether there was clonal variability in the potential for HeLa clones to support LINE-1 retrotransposition, we performed LINE-1 retrotransposition assays using eight individual HeLa cell clones and the parental population of HeLa from which they originated. The results of the LINE-1 retrotransposition assays in HeLa clones confirm that some cell clones are nearly incapable of supporting LINE-1 retrotransposition (HeLa clone 1), while other cell clones are particularly amenable to LINE-1 retrotransposition events (HeLa clone 7). LINE-1 retrotransposition rates in HeLa clone 7 (mean = 699 colonies) were significantly increased compared to those in HeLa clone 1 (mean = 5 colonies) (Figure 1A). Both HeLa clone 1 and HeLa clone 7 differ significantly from the parental HeLa population in their ability to undergo LINE-1 retrotransposition, with the parental population showing a level of LINE-1 retrotransposition intermediate between the two clones (Figure 1A). Representative flask images for HeLa clones are shown in Additional file 1: Figure S1A,B.

To test if the large discrepancy in LINE-1 retrotransposition potential between HeLa clones 1 and 7 was paralleled for Alu retrotransposition, we performed Alu retrotransposition assays in the same HeLa clones. As was the case with LINE-1 retrotransposition, the ability of HeLa clone 7 to retrotranspose Alu (mean = 503 colonies) was significantly elevated (252-fold) compared to the ability of HeLa clone 1 to support Alu retrotransposition (mean = 1 colony). Additionally, remaining HeLa subclones were fairly consistent in their ability to retrotranspose Alu, showing fairly modest rates of retrotransposition. None of the individual HeLa clones supported Alu retrotransposition as well as the parental population, suggesting that there was even more heterogeneity that was not sampled in this study (Figure 1B).

To test if the observed clonal effect on LINE-1 and Alu retrotransposition was specific to HeLa cells, we tested LINE-1 and Alu retrotransposition in clones of HCT116 cells, as above. Unlike HeLa clones, HCT116 clones did not exhibit any significant variation in either LINE-1 or Alu retrotransposition rates in any of the tested clones (Figure 1C,D). Additionally, the parental population of HCT116 cells showed similar levels of retrotransposition to each of the clones (Figure 1C,D). This is in contrast to our HeLa data, which showed a 140-fold and 503-fold difference between retrotransposition permissive and non-retrotransposition permissive clones for LINE-1 and Alu, respectively (Figure 1A,B). Representative flask images for HCT116 clones are shown in Additional file 1: Figure S1C,D.

**Figure 2:**
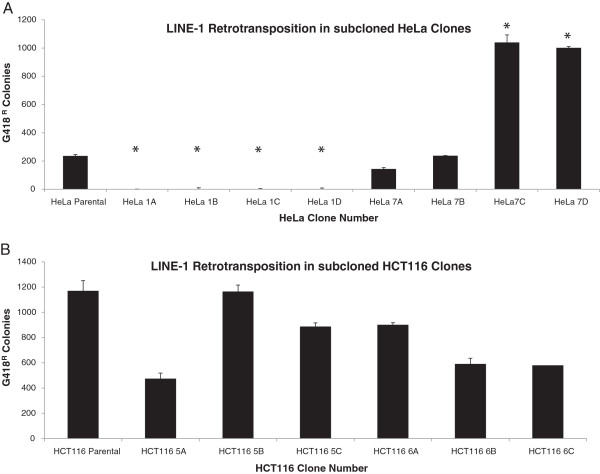
**Differences in LINE-1 retrotransposition between cell clones are maintained upon cell subcloning. (A)** HeLa clones 1 and 7 were re-cloned and LINE-1 retrotransposition assays were performed. The differences in LINE-1 retrotransposition potential were maintained between HeLa subclones, with the four HeLa clone 1 subclones exhibiting a large reduction in LINE-1 retrotransposition rates compared to the four HeLa clone 7 subclones. **(B)** HCT116 clones 5 and 6 were re-cloned and LINE-1 retrotransposition assays were performed. As with the original HCT116 cell clones, there were no significant differences in LINE-1 retrotransposition rates between HCT116 subclones. Error bars represent SEM. Sample size is four independent experiments (independent transfections) each performed in triplicate (n = 12). Asterisks indicate statistical significance from the parental population of at least *P* <0.05 by one-way ANOVA with Tukey’s post-test. LINE-1, long interspersed element-1; SEM, standard error of the mean.

We next wanted to determine if the observed differences in LINE-1 and Alu retrotransposition in clones of HeLa when compared to HCT116 clones was stable upon subcloning. This scenario is an experimental mimic to what might occur during tissue culture passaging if any one cell outgrows the others to become the predominant component of the cell mixture. To this end, we re-cloned two of the original HeLa clones that showed varying degrees of support for retrotransposition of LINE-1 and Alu (clones 1 and 7) to obtain HeLa subclones 1A, 1B, 1C, 1D and 7A, 7B, 7C and 7D. We also subcloned two HCT116 clones (clones 5 and 6) to obtain HCT116 subclones 5A, 5B, 5C and 6A, 6B and 6C. We then performed the same LINE-1 retrotransposition assay as above on the HeLa and HCT116 subclones and the parental populations of cells. The LINE-1 retrotransposition differences seen in the re-cloned HeLa clones (1A, 1B, 1C, 1D and 7A, 7B, 7C and 7D) was consistent with the observed difference in these two clones prior to re-cloning (compare Figure 2A to Figure 1A) in that the subclones of HeLa clone 1 all remained essentially incapable of undergoing retrotransposition. It is interesting to note, however, that we observed variability in the rates of retrotransposition in the subclones of HeLa clone 7, with two of the subclones (HeLa subclones 7A and 7B) displaying significantly reduced rates of LINE-1 retrotransposition when compared to two others (HeLa subclones 7C and 7D) (Figure 2A). Similarly in HCT116 clones 5A, 5B, 5C and 6A, 6B and 6C, there was no statistically significant observed difference in the rates of LINE-1 retrotransposition between clones (Figure 2B), which is in agreement with the data from these cells before being re-cloned (Figure 1B). Taken together, these data imply that the observed clonal differences in ability to support retrotransposition are stable to varying extents and are propagated as cells are passaged.

## Discussion

Our data demonstrate that differences in the rates of LINE-1 and Alu retrotransposition between different populations of the same cell type may be the result of clonal variation in retrotransposition potential being accumulated over time. While no noticeable differences were observed in growth rate or morphology between clones, it is possible that this clonal variation is the result of genetic differences between cell clones that arose in the culture. Additionally, it is possible that these results are explained by a potentially more reversible epigenetic change. However, the implications of variations in retrotransposition competency between clones of the same cell type are important to consider in many experimental designs. n a mixed population of cells, any one individual cell has the potential to outgrow other cells in tissue culture. If this is the case, this individual cell’s ability to support retrotransposition predominates in the mixed population of cells. This can result in a population of cells becoming either increasingly supportive of retrotransposition or losing the ability to support retrotransposition altogether. In addition, if an experimental protocol involves cell cloning, as is often the case when using shRNA and other genetic transformations, one must consider the arbitrary nature of this potential cloning variation. Thus, it may be worth characterizing multiple cell clones, or alternatively subcloning a cell line prior to such studies in order to begin with a more homogeneous cell population.

It seems obvious that due to their potential to cause mutagenic (reviewed in [3,4,9]) and toxic [4,10] effects on cells, organisms have developed a remarkably wide array of defenses against mobile elements. These have included a number of factors that suppress transcription (reviewed in [11]), as well as influences thought to be at the level of the RNA [11-13] or even the integration process [14,15]. Thus, because so many factors control retrotransposition, there are many genetic variations that can occur to cells that may alter the process. Our data show that simply culturing cells over long periods of time result in variations in factors that influence retrotransposition. This may have particular relevance to cancer where cells generate genetic diversity rapidly and new clonal variants arise during tumor progression. This clonal variation may be one of the reasons why there is so much variation in L1 activity between different types of cancer, as well as between different cancers of the same type [16,17].

## Abbreviations

ANOVA: Analysis of variance; FBS: Fetal bovine serum; LINE-1: Long interspersed element-1; non-LTR: Non-long terminal repeat; MEM: Minimum essential medium; SD: Standard deviation.

## Competing interests

The authors declare that they have no competing interests.

## Authors’ contributions

VAS and ZJF designed and performed experiments. VAS wrote the manuscript. PLD conceived and designed experiments and edited the manuscript. All authors read and approved the final manuscript.

## Supplementary Material

Additional file 1 **Representative flask images of HeLa and HCT116 clones. (A)** Alu and LINE-1 retrotransposition and pIRES colony formation in HeLa clone 7. **(B)** Alu and LINE-1 retrotransposition and pIRES colony formation in HeLa clone 1. **(C)** Alu and LINE-1 retrotransposition and pIRES colony formation in HCT116 clone 7. **(D)** Alu and LINE-1 retrotransposition and pIRES colony formation in HCT116 clone 3. Click here for file
