# Supplementary figures and images for: LINE-1 and Alu retrotransposition exhibit clonal variation
Source: Mob DNA. 2013 Jun 3;4:16. doi: 10.1186/1759-8753-4-16 (PMC3716877; doi:10.1186/1759-8753-4-16)

# Supplementary Figure 1

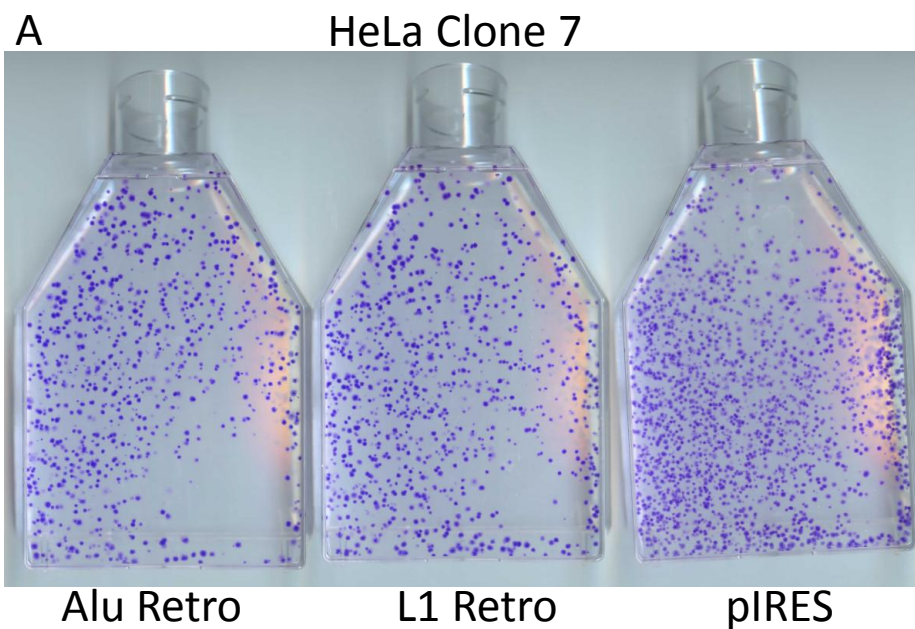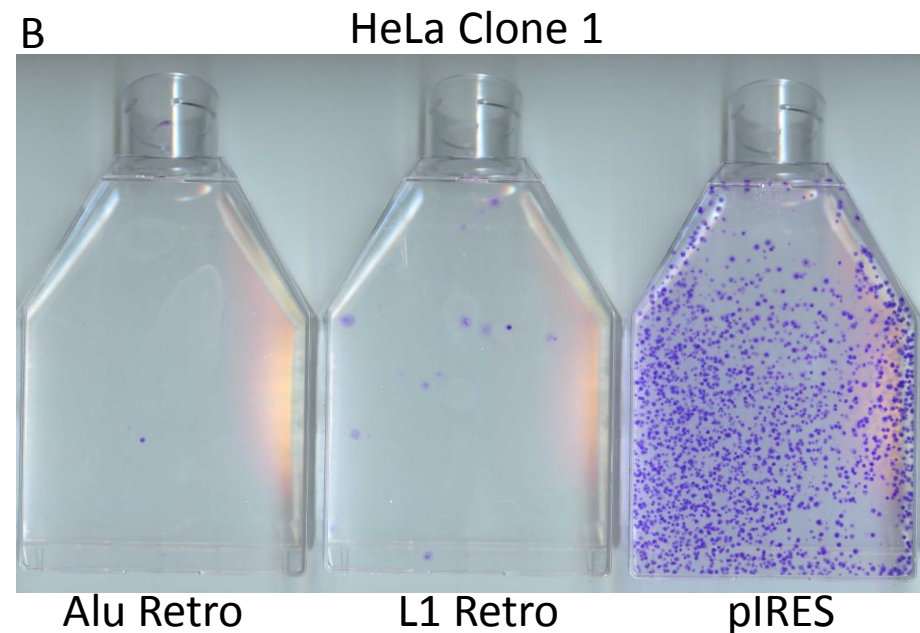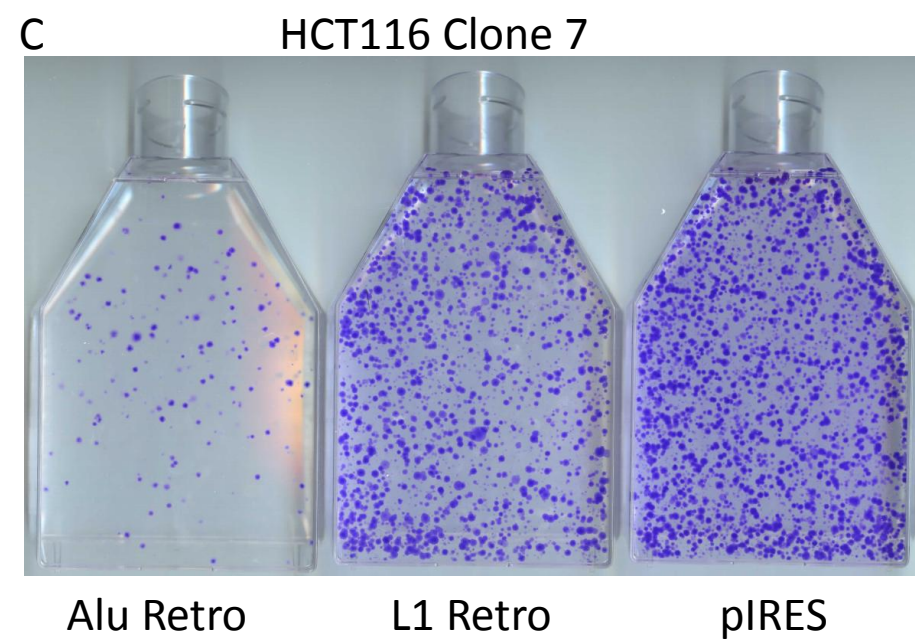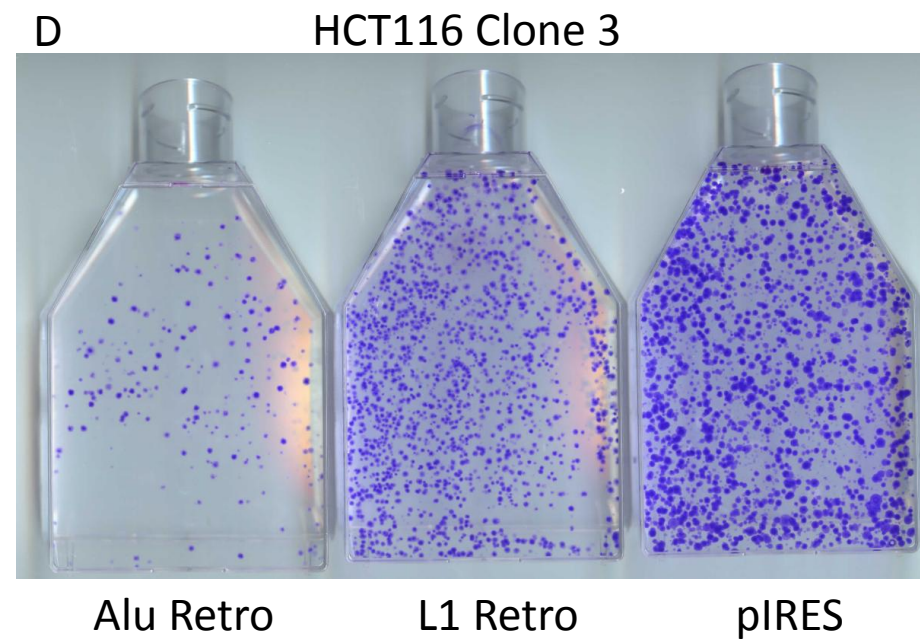

Supplement: Additional file 1 — Representative flask images of HeLa and HCT116 clones. (A) Alu and LINE-1 retrotransposition and pIRES colony formation in HeLa clone 7. (B) Alu and LINE-1 retrotransposition and pIRES colony formation in HeLa clone 1. (C) Alu and LINE-1 retrotransposition and pIRES colony formation in HCT116 clone 7. (D) Alu and LINE-1 retrotransposition and pIRES colony formation in HCT116 clone 3. [file 1759-8753-4-16-S1.pdf]
